# Clinical implication of quantitative flow ratio to predict clinical events after drug‐coated balloon angioplasty in patients with in‐stent restenosis

**DOI:** 10.1002/clc.23630

**Published:** 2021-05-19

**Authors:** Jiani Tang, Hanjing Hou, Jiapeng Chu, Fei Chen, Yian Yao, Yanhua Gao, Zi Ye, Shaowei Zhuang, Yan Lai, Xuebo Liu

**Affiliations:** ^1^ Department of Cardiology Tongji Hospital, Tongji University School of Medicine Shanghai China; ^2^ Department of Cardiology Seventh People's Hospital, Shanghai University of Traditional Chinese Medicine Shanghai China

**Keywords:** drug‐coated balloon, percutaneous coronary intervention, quantitative flow ratio

## Abstract

**Background:**

The association between the quantitative flow ratio (QFR) and adverse events after drug‐coated balloon (DCB) angioplasty for in‐stent restenosis (ISR) lesions has not been investigated.

**Hypothesis:**

Post‐procedural QFR is related to adverse events in patients undergoing DCB angioplasty for ISR lesions.

**Methods:**

This retrospective study included data from patients undergoing DCB angioplasty for drug‐eluting stent (DES) ISR between January 2016 and February 2019. The QFR was measured at baseline and after DCB angioplasty. The endpoint was the vessel‐oriented composite endpoint (VOCE), defined as a composite of cardiac death, vessel‐related myocardial infarction, and ischemia‐driven target vessel revascularization.

**Results:**

Overall, 177 patients with 185 DES‐ISR lesions were included. During 1‐year follow‐up, 27 VOCEs occurred in 26 patients. The area under curve (AUC) of the post‐procedural QFR was statistically greater than that of the in‐stent percent diameter stenosis (0.77, 95% confidence interval [CI] 0.67–0.87 vs. 0.64, 95% CI 0.53–0.75; *p* = .032). Final QFR cutoff of 0.94 has the best predictive accuracy for VOCE. A QFR > 0.94 was associated with a lower risk of VOCE compared to a QFR ≤ 0.94 (log‐rank test, *p* < .0001). Survival analysis using the multivariable Cox model showed that a post‐procedural QFR ≤ 0.94 was an independent predictor of 1‐year VOCE (hazard ratio 6.53, 95% CI 2.70–15.8, *p* < .001).

**Conclusions:**

A lower QFR value was associated with worse clinical outcomes at 1 year after DCB angioplasty for DES‐ISR.

## 1 INTRODUCTION

Although drug‐eluting stents (DES) effectively inhibit neointimal proliferation and markedly reduce the incidence of in‐stent restenosis (ISR),^1^, ^2^ recurrent ISR requiring repeat revascularization still occurs after DES implantation,^3^ and treatment of DES‐ISR remains a major challenge in the percutaneous coronary intervention (PCI) field. Drug‐coated balloons (DCB) are semi‐compliant balloons covered with anti‐proliferative drugs such as lipophilic paclitaxel and have been proposed as an alternative to DES.^4^, ^5^ During balloon inflation, the lipophilic paclitaxel is delivered to the vessel wall surface providing an antiproliferative effect and preventing neointimal hyperplasia without additional metallic layers. In this respect, several clinical trials support the efficacy of DCB in the treatment of ISR.^6^, ^7^, ^8^

The fractional flow reserve (FFR) is the gold standard to assess the physiological severity of coronary stenosis.^9^ Several trials have reported an inverse relationship between the post‐interventional FFR and the risk of subsequent adverse events.^10^, ^11^ Additionally, the FFR has been identified as a reference standard to ascertain functional ISR severity.^12^ Quantitative flow ratio (QFR), a novel technique for the rapid computation of FFR from coronary angiography without the use of pressure wires or hyperemic agents, has good correlation with FFR and proven clinical value in guiding pre‐ and post‐PCI management.^13^, ^14^, ^15^ Although the diagnostic performance of the QFR in assessing ISR lesions using FFR as a reference standard has been recently investigated,^16^ its utility in the context of DCB angioplasty remains unknown. In the present study, we aimed to perform the first validation of the QFR as a tool to predict events in patients with ISR treated with DCB.

## 2 MATERIALS AND METHODS

### 2.1 Study design and population

This was a retrospective analysis of consecutive patients with at least one ISR lesion who underwent DCB angioplasty between January 2016 and February 2019 from two centers in Shanghai, China. Exclusion criteria were a TIMI flow grade <3 at baseline or after DCB angioplasty, ST‐segment elevation myocardial infarction (MI), major procedural complications requiring DES implantation, and significant left main ISR lesion or inadequate angiographic image quality limiting the QFR computation. The study protocol complied with the Declaration of Helsinki and was approved by the ethics committee at each participating institution.

### 2.2 PCI procedures and angiographic characteristics

ISR lesions were treated using paclitaxel‐coated balloons (Sequent Please, B Braun Melungeon, Germany). All patients were pretreated with aspirin and a P2Y12 inhibitor (i.e., clopidogrel or ticagrelor). The DCB size was selected based on the length of the target lesion and the diameter of the previously used stents. The details of the PCI strategy and intravascular imaging utilization were left entirely at the operators' discretion. The ISR was classified as focal (type I, <10 mm in length), diffuse (type II, >10 mm in length) and proliferative (type III, >10 mm in length and extending outside the stent).^17^

Quantitative coronary angiography analyses were performed before and after DCB angioplasty using an offline computerized quantitative coronary angiographic system (CAAS system; Pie Medical Instruments, Maastricht, The Netherlands). The following parameters were measured: reference vessel diameter (RVD), minimal lumen diameter (MLD), and percent diameter stenosis (%DS). Measurements of the stented area were obtained shoulder‐to‐shoulder (in‐stent) and including the total stented area plus 5 mm proximally and distally (in‐segment). The acute lumen gain was calculated as the difference between post‐ and pre‐ procedural MLD.

### 2.3 Off‐line QFR assessment

**FIGURE 1:**
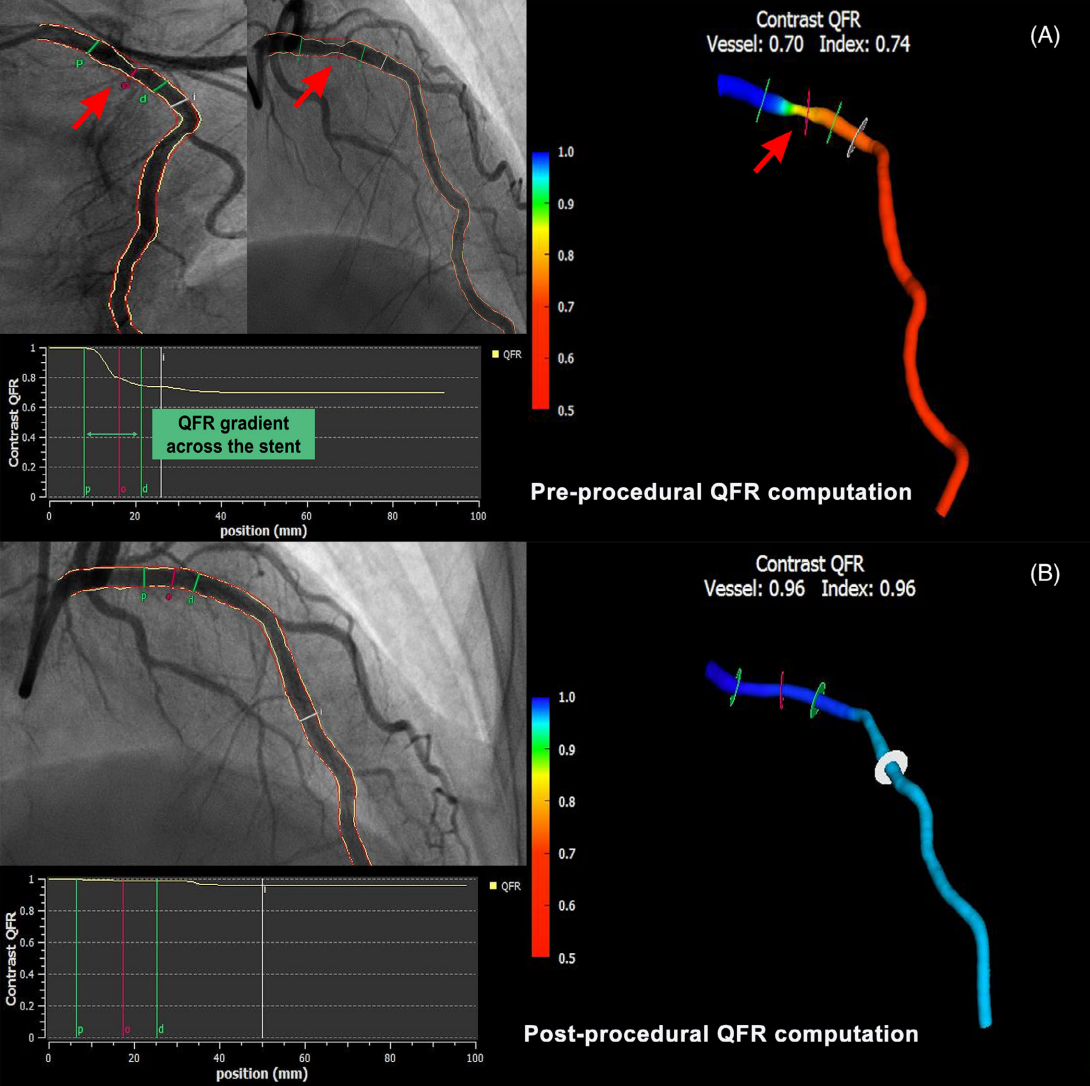
Case example of reconstructed 3‐D QCA and measured QFR. QFR calculation was based on the 3D‐QCA reconstructed from two angiographic projections with angles ≥25 ° apart and 3D reconstruction of the interrogated vessel without its side branches was performed. (A) Pre‐procedural angiographic image shows a ISR lesion, and QFR was 0.70 (B) Final angiography showed minimal residual stenosis after DCB treatment, and QFR was 0.96. Red arrows indicate the target ISR lesion. DCB, drug‐coated balloon; ISR, in‐stent restenosis; QCA, quantitative coronary angiography; QFR, quantitative flow ratio

Off‐line QFR was performed by experienced analysts certified for the use of the QFR system software (AngioPlus, Pulse Medical Imaging Technology, Shanghai Co. Ltd., Shanghai, China). For QFR computations, two angiographic projections, at least 25° apart, were transferred to the QFR system, and three‐dimensional reconstruction of the interrogated vessel without its side branches was performed as described elsewhere.^13^, ^18^, ^19^ The lumen contour was estimated automatically. Manual correction was performed in cases of suboptimal angiographic image quality based on a standard operation procedure. A contrast flow model incorporating contrast flow velocity based on the frame count method was used for QFR computations. Three‐dimensional quantitative coronary analysis data were readily available. Subsequently, the QFR was computed. Case example is provided in Figure 1.

### 2.4 Follow‐up and outcome definition

In the present study, we investigated the relationship between the post‐procedural QFR and clinical outcomes at the vessel level. Clinical follow‐up data derived from clinical visits, telephone interviews, and from hospital records of any readmissions.

The primary endpoint of this study was the vessel‐oriented composite endpoint (VOCE) at 1 year, defined as the composite of cardiac death, vessel‐related MI, or ischemia‐driven target vessel revascularization (TVR). The secondary endpoints were the individual components of the VOCE.

Any death of unknown cause was assumed as due to cardiac cause. The diagnosis of MI was based on the fourth universal definition of MI, which requires a combination of symptoms, electrocardiographic changes, and significant increase in troponin.^20^ When the identification of the culprit vessel was not possible, the endpoint was evaluated considering each vessel treated with DCB. Ischemia‐driven TVR was defined as any repeated revascularization of the target vessel by either PCI or coronary artery bypass grafting (CABG) in the presence of a lesion with a %DS >50%, and with at least one of the following: (i) recurrence of angina, (ii) positive non‐invasive test, and (iii) positive invasive physiologic test. All angiograms of patients who underwent TVR were reviewed to identify the target lesion revascularization.

### 2.5 Statistical analysis

Continuous variables are presented as the median with interquartile range and compared between groups using the Mann–Whitney *U* test. Categorical variables are summarized as frequencies and proportions and compared using Pearson's chi‐square or Fisher's exact test. Intraclass correlation coefficient analysis was performed to evaluate inter‐ and intra‐observer agreement. Receiver operator characteristic curve (ROC) analysis was performed to determine the optimal post‐procedural QFR cut‐off value to predict VOCE occurrence. ROC and the area under curve (AUC) were performed to assess and compare the ability of the post‐procedural QFR and in‐stent %DS for predicting adverse outcomes using the DeLong method.^21^ Cox multivariable analysis was performed to determine independent predictors of the VOCE. We selected the variables by means of the LASSO (Least Absolute Shrinkage and Selection Operator) method.^22^ Hazard ratios (HRs) with corresponding 95% confidence interval (CI) were provided. The Kaplan–Meier method was used to demonstrate the timing of events during follow‐up in relation to the post‐procedural QFR value, and the log‐rank test was applied to compare survival curves among groups. A significant level was defined when *p* < .05. All analyses were performed using R software, version 3.4.3. (R Foundation for Statistical Computing, Vienna, Austria).

## 3 RESULTS

### 3.1 Patient characteristics

The flow chart of this study was shown in Figure S1. During the study period, a total of 177 patients with 185 DES‐ISR lesions undergoing DCB angioplasty were eligible for inclusion in this study. The baseline patient characteristics are outlined in Table S1. The median age was 68 (62–75) years and male sex was predominant (81%). The median interval from the latest DES implantation to the DCB angioplasty for ISR lesion was 389 (230–1725) days. Apart from a higher rate of diabetes mellitus in the VOCE group (69.2% vs. 43.7%, *p* = .016), there were no significant differences in age, gender, clinical risk factors, laboratory results and clinical diagnosis between patients with or without VOCE.

Characteristics of the lesions and procedures are shown in Table 1. Of 185 vessels evaluated, 93 (50.3%) corresponded to left anterior descending arteries, 37 (20%) to left circumflex arteries, and 55 (29.7%) to righ coronary artries. There were no significant differences in the index DES type, lesion characteristics, restenosis classification, or rate of edge‐ISR. The pre‐procedural lesion lengths, RVD, MLD, %DS, and rate of cutting balloon use or DCB use beyond 1 were similar in both groups.

**TABLE 1:** Characteristics of lesions and procedures (n = 185)

	VOCE	Non‐VOCE	*p* value
	(n = 27)	(n = 158)	
Target artery			.077
Left anterior descending, n (%)	19 (70.4)	74 (46.8)	
Left circumflex, n (%)	3 (11.1)	34 (21.5)	
Right coronary, n (%)	5 (18.5)	50 (31.6)	
Index stent type			
Everolimus‐eluting, n (%)	9 (33.3)	36 (22.8)	.238
Sirolimus‐eluting, n (%)	11 (40.7)	89 (56.3)	.133
Zotarolimus‐eluting, n (%)	5 (18.5)	21 (13.3)	.673
Unknown, n (%)	2 (7.4)	12 (7.6)	>.999
Moderate or severe calcification, n (%)	5 (18.5)	18 (11.4)	.471
Bifurcation lesion, n (%)	4 (14.8)	40 (25.3)	.236
>1 intervention on target lesion, n (%)	3 (11.1)	18 (11.4)	>.999
IVUS use, n (%)	6 (22.2)	34 (21.5)	.936
Restenosis classification			
Focal, n (%)	11 (40.7)	65 (41.1)	.969
Diffuse, n (%)	9 (33.3)	61 (38.6)	.602
Proliferative, n (%)	7 (25.9)	32 (20.3)	.504
Edge‐ISR, n (%)	2 (7.4)	20 (12.7)	.747
Pre‐procedural QCA			
Reference vessel diameter, mm	2.7 (2.4–2.8)	2.7 (2.4–3.0)	.325
Minimal lumen diameter, mm	0.9 (0.7–1.1)	0.9 (0.7–1.2)	.672
Diameter stenosis, %	64.0 (56.0–73.4)	66.7 (56.1–75.0)	.843
Lesion length, mm	20.0 (10.4–31.0)	18.6 (10.0–24.9)	.227
Post‐procedural QCA (in‐segment)			
Minimal lumen diameter, mm	1.6 (1.5–2.0)	1.9 (1.8–2.2)	*.008*
Diameter stenosis, %	34.5 (30.9–40.0)	28.3 (23.4–34.2)	*.003*
Acute lumen gain, mm	0.8 (0.6–1.1)	1.0 (0.7–1.3)	*.021*
Post‐procedural QCA (in‐stent)			
Minimal lumen diameter, mm	1.7 (1.5–2.1)	2.0 (1.8–2.2)	*.017*
Diameter stenosis, %	31.0 (25.9–36.0)	25.1 (21.4–32.2)	*.018*
Acute lumen gain, mm	0.9 (0.7–1.2)	1.0 (0.8–1.3)	.238
Physiological results			
Pre‐procedural QFR	0.67 (0.52–0.76)	0.71 (0.61–0.78)	.084
Pre‐procedural QFR > 0.80, n (%)	0 (0.0)	6 (3.8)	.595
Post‐procedural QFR	0.91 (0.83–0.95)	0.98 (0.95–0.99)	*<.001*
Post‐procedural QFR ≤0.80, n (%)	5 (18.5)	6 (3.8)	*.011*
Balloon pre‐dilation	27 (100)	158 (100)	>.999
Diameter, mm	3.0 (2.5–3.0)	2.75 (2.5–3.0)	.550
Length, mm	15 (15–15)	15 (15–15)	.935
Pressure, atm	15 (14–16)	14 (12–18)	.934
Inflation time, s	5 (5–10)	5 (5–10)	.767
Cutting balloon	3 (11.1)	33 (20.9)	.236
Diameter, mm	2.8 (2.6–2.9)	3.0 (2.5–3.0)	.583
Length, mm	10 (10–11.5)	10 (10–10)	.650
Pressure, atm	8 (8–10)	10 (8–12)	.678
Inflation time, s	5 (5–5)	5 (5–10)	.303
DCB			
No. of DCB used >1	3 (11.1)	14 (8.9)	.719
Diameter, mm	3.0 (2.6–3.0)	3.0 (2.5–3.0)	.953
Length, mm	26 (20–28)	20 (20–26)	.703
Inflation pressure, atm	8 (7–8)	8 (7–8.75)	.796
Inflation time, s	60 (60–60)	60 (60–60)	.553
Balloon‐to‐artery ratio	1.08 (1.05–1.15)	1.10 (0.97–1.19)	.930

*Note*: Values are shown as median (25th–75th percentile) or number (%). *p* values < .05 are in italics.

Abbreviations: DCB, drug‐coated balloon; ISR, in‐stent restenosis; IVUS, intravascular ultrasound; QCA, quantitative coronary angiography; QFR, quantitative flow ratio.

Regarding post‐procedural changes, while the acute lumen gain of the in‐stent segment behaved similarly between groups, the %DS and MLD were significantly higher and smaller, respectively, in vessels with VOCE than in vessels without VOCE (31.0% [25.9%–36.0%] vs. 25.1% [21.4%–32.2%], *p* = .018; 1.7 mm [1.5–2.1 mm] vs. 2.0 mm [1.8–2.2 mm], *p* = .017; respectively). With respect to the in‐segment post‐procedural quantitative coronary angiography analysis, vessels with VOCE showed a significantly larger %DS (34.5% [30.9%–40.0%] vs. 28.3% [23.4%–34.2%], *p* = .003), smaller MLD (1.6 mm [1.5–2.0 mm] vs. 1.9 mm [1.8–2.2 mm], *p* = .008), and smaller acute lumen gain (0.8 mm [0.6–1.1 mm] vs. 1.0 mm [0.7–1.3 mm], *p* = .021) than vessels without VOCE.

Before and after DCB angioplasty, the intraclass correlation coefficients for inter‐observer variability of QFR was 0.93 (95% CI, 0.91–0.95) and 0.94 (95% CI, 0.92–0.95), respectively. Agreement in QFR was also excellent for the same analysts (Pre‐procedure: 0.95 [95% CI, 0.92–0.97] and post‐procedure: 0.94 [95% CI, 0.92–0.96]).

The distribution of individual QFRs before and after DCB angioplasty is reported in Figure S2A. While there was no significant difference between the two groups with respect to the pre‐procedural QFR (0.67 [0.52–0.76] vs. 0.71 [0.61–0.78], *p* = .084), vessels with VOCE had a significantly lower post‐procedural QFR than vessels without VOCE (0.91 [0.83–0.95] vs. 0.98 [0.95–0.99], *p* < .001).

### 3.2 Definition of a potential cutoff value

**FIGURE 2:**
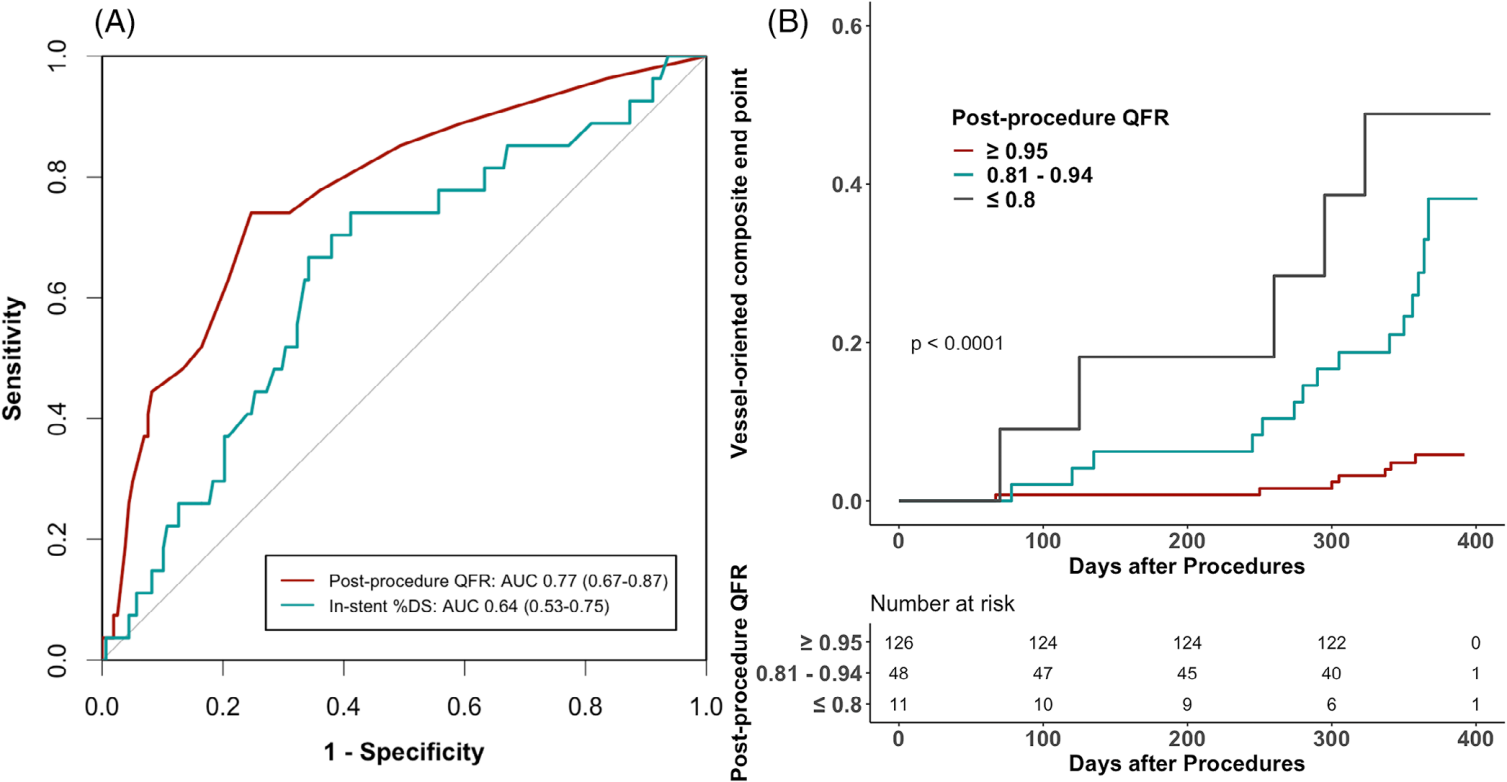
**(**A) Receiver operator characteristic curves for the VOCE. The red line corresponds to the QFR value (AUC 0.77, 95% CI 0.67–0.87) and the green line to the in‐stent %DS (AUC 0.64, 95% CI 0.53–0.75). (B) Kaplan–Meier curves of VOCE occurrence at 1‐year follow‐up according to the post‐procedural QFR. AUC, area under curve; CI, confidence interval; %DS, percent diameter stenosis; QFR, quantitative flow ratio; VOCE, vessel‐oriented composite endpoint

The occurrence of the VOCE stratified according to the post‐procedural QFR is depicted in Figure S2B. ROC analysis showed that the optimal post‐procedural QFR cut‐off value for predicting the VOCE was 0.94, with a sensitivity of 74% and specificity of 75%. The ROC curves for the in‐stent %DS and post‐procedural QFR are shown in Figure 2(A). In the direct comparison with in‐stent %DS, QFR showed better ability to discriminate vessels at risk of the VOCE (AUCs 0.77 [95% CI 0.67–0.87] vs. 0.64 [95% CI 0.53–0.75], *p* = .032).

### 3.3 Clinical outcomes

Clinical follow‐up was completed in all patients. The median patient follow‐up was 364 days (355–369 days). Clinical outcomes at 12 months are summarized in Table 2. Patients who achieved a post‐procedural QFR > 0.94 had significantly less VOCEs than those with values ≤0.94 (5.6% vs. 33.9%, *p* < .001); this difference was mostly driven by a higher rate of TVR (4.8% vs. 32.2%, *p* < .001). Two patients presented more than one event during the follow‐up; both developed target vessel MI and underwent target lesion revascularization. One patient with a post‐procedural QFR ≤ 0.94 died of cardiac cause. No cardiac death occurred in patient with a post‐procedural QFR > 0.94.

**TABLE 2:** Clinical outcomes stratified by the cutoff value of post‐procedure QFR at vessel level

	QFR ≤ 0.94	QFR > 0.94	*p* value
	(n = 59)	(n = 126)	
Death	2 (3.4)	1 (0.8)	.491
Cardiac death	1 (1.7)	0 (0.0)	>.999
Myocardial infarction	3 (5.1)	1 (0.8)	.072
Target vessel MI	2 (3.4)	1 (0.8)	.179
Target vessel revascularization	19 (32.2)	6 (4.8)	*<.001*
Coronary angioplasty	17 (28.8)	5 (4.0)	*<.001*
Coronary surgery	2 (3.4)	1 (0.8)	.317
Target lesion revascularization	18 (30.5)	6 (4.8)	*<.001*
VOCE	20 (33.9)	7 (5.6)	*<.001*

*Note*: patients with >1 event are counted only once for the composite clinical endpoint, although each event is listed separately in the corresponding category. *p* values < .05 are in italics.

Abbreviations: MI, myocardial infarction; QFR, quantitative flow ratio; VOCE, vessel‐oriented composite endpoint.

Repeat revascularization was performed in 19 (32.2%) vessels with a post‐procedural QFR ≤0.94 due to recurrent ISR; two of these cases underwent CABG. In turn, six (4.8%) vessels with final QFR > 0.94 required reinterventions: five underwent coronary stenting and one CABG. The Kaplan–Meier curves depicting the occurrence of the VOCE within the study period are shown in Figure 2(B). Lower post‐procedural QFR values were significantly associated with a higher incidence of the VOCE (*p* < .0001).

Variables predicting VOCE obtained after penalization using LASSO method included following three variables: post‐procedural QFR ≤0.94; diabetes mellitus; post‐procedural in‐stent %DS. Multivariable Cox proportional hazards analyses revealed that, after adjusting for potential confounding factors, a post‐procedural QFR of ≤0.94 was associated with an over 6‐fold increase in the risk of the VOCE (hazard ratio [HR] 6.53, 95% CI 2.70–15.8; *p* < .0001) (Table 3 **)**. When considering the post‐procedural QFR as a continuous variable, post‐procedural QFR (each 0.1 increase) was associated with lower risk of VOCE in multivariable adjusted analysis (HR 0.36; 95% CI 0.22–0.59, *p* < .001) (Table 3).

**TABLE 3:** Multivariable cox regression analyses for predictors of VOCE (n = 27)

	HR (95% Cl)	p value
Model 1		
Post‐procedural QFR ≤0.94	6.53 (2.70–15.8)	*< .001*
Diabetes mellitus	2.32 (1.04–5.19)	*.040*
Diameter stenosis (Post‐procedural in‐stent)	1.03 (0.99–1.07)	.196
Model 2		
Post‐procedural QFR (per 0.1 increase)	0.36 (0.22–0.59)	*< .001*
Diabetes mellitus	1.76 (0.77–4.05)	.180
Diameter stenosis (Post‐procedural in‐stent)	1.02 (0.99–1.07)	.124

*Note*: Models after LASSO (Least Absolute Shrinkage and Selection Operator) variable selection method. *p* values < .05 are in italics.

Abbreviations: CI, confidence interval; HR, hazard ratio; QFR, quantitative flow ratio; VOCE, vessel‐oriented composite endpoint.

## 4 DISCUSSION

The main findings of this study are as follows: (1) a low QFR after DCB angioplasty in DES‐ISR lesions was an independent predictor of adverse clinical events during a 1‐year follow up; (2) compared with the in‐stent %DS, the QFR has a better ability to predict vessel‐related clinical outcomes after DCB angioplasty.

To the best of our knowledge, this is the first study to describe the utility of the QFR to predict clinical events after DCB angioplasty for ISR lesions. Our results showed that the QFR measured immediately after the procedure had an inverse relationship with future clinical events. Notably, the pre‐procedure QFR had no association with outcomes. Conceptually, interventions destined to increase the post‐procedural QFR may be able to improve long‐term outcomes.

Although the choice of the PCI strategy has routinely been based on angiographic findings, these have limited efficacy to predict immediate physiological results or clinical outcomes of coronary stenting.^23^ As a result, the concept of functional optimization of PCI results has been explored for a long time. In this regard, a previous study found a graded relationship between post‐PCI FFR and major adverse cardiovascular events.^24^ With respect to analyses of the final functional results after DES implantation, a large meta‐analysis performed by Johnson et al^25^ showed an inverse relationship between post‐PCI FFR and the rate of vessel‐related adverse outcomes. These results suggest that post‐PCI physiological assessments can predict adverse clinical outcomes by identifying residual disease across the stented and adjacent segments. For the determination of procedural success, angiographic residual %DS has been commonly used in clinical practice, but its limitation is well known. In this study, we compared the prognostic ability of QFR and in‐stent stenosis severity after DCB angioplasty on clinical events, and we found that the former had a better performance.

The optimal treatment strategy for DES‐ISR remains undefined. Based on clinical trials that support its efficacy, angioplasty with DCB is recommended for the treatment of ISR in the European clinical practice guidelines (Class I, Level of Evidence: A).^26^ Prospective studies have confirmed the utility of the FFR to guide clinical decision making in ISR treatment and suggest that revascularization can be safely deferred in patients with an FFR > 0.75.^27^ Recent studies found that FFR‐guided DCB treatment of de novo lesions appeared feasible and safe in stable patients.^28^ Whether the use of post‐procedural physiological assessments could be expanded to angioplasty with DCB for ISR lesions had not been sufficiently studied to date.

Nevertheless, while evidence supporting the utility of FFR has been mounting, FFR is still largely underutilized in clinical practice.^29^ Reasons for this may include the high equipment and drug costs, and the risk of related complications. Progress in angiography‐derived FFR such as the QFR can reduce these limitations by calculation of functional parameters in a simpler and rapid way. A recent study demonstrated substantial applicability of the QFR in functional assessment of ISR lesions, using FFR as reference standard.^16^ Furthermore, Li et al.^30^ investigated the functional results following DCB or DES treatment in small‐vessel disease and demonstrated that assessing the QFR in small coronary arteries was feasible. Therefore, these studies suggest that the QFR is not only a promising tool in assessing clinical results of stenting but can also be applied to assess the efficacy of DCB in the treatment of ISR and de novo lesions.

Unlike pre‐procedural functional assessment, where parameters to determine an ischemia‐causing stenosis are clearly defined, post‐procedural cutoff values vary widely due to multiple factors, including the population studied, lesion and procedural characteristics, the presence of multivessel disease and the incidence of clinical events.^11^, ^12^, ^14^ Recently, in the HAWKEYE study, Biscaglia et al.^14^ reported that post‐PCI QFR lower than 0.90 was associated with a higher rate of VOCE. Kogame et al.^15^ demonstrated that the cutoff value of post‐PCI QFR was 0.91 in relatively high‐risk patients with de novo 3‐vessel disease. Our previous study found that post‐PCI QFR ≥0.91 was associated with a lower rate of VOCE.^31^ In our present study, we were able to identify a threshold for post‐procedural QFR (0.94) that could be used to discriminate ISR lesions treated by DCB angioplasty at a higher risk of clinical events. In addition to the utility of this dichotomous approach, results of Cox proportional hazards regression analysis revealed that progressive decreases in the QFR were also related to a higher risk of adverse clinical outcomes at 1‐year follow‐up.

There are a number of limitations in the current study. First, the small sample size precluded subgroup analysis. Second, the rigorous inclusion and exclusion criteria have theoretically introduced a selection bias; thus, our conclusions cannot be extrapolated to the excluded patients. Third, the determination of the threshold value was based solely on our clinical data; the optimal values may vary by different populations. Additional studies are needed to validate the cutoff value derived in our study. Finally, although a lower QFR was associated with a higher rate of adverse outcomes, this study was not able to evaluate the clinical impact of interventions to improve suboptimal post‐procedural functional results. Large randomized controlled trials are necessary to address this question.

## 5 CONCLUSION

A low post‐procedural QFR was associated with poor clinical outcomes within 1 year after DCB angioplasty for DES‐ISR. Compared with the post‐procedural in‐stent %DS, the QFR may be superior to predict vessel‐related clinical outcomes. Future studies should be conducted to confirm our results and evaluate the utility of the QFR to guide therapeutic decisions in ISR lesions.

## CONFLICT OF INTEREST

The authors have no potential conflict of interest.

## Supporting information


**Table S1** Baseline patient characteristics (n = 177)
**Figure S1** Flow chart of patient selection. DCB, drug‐coated balloon; ISR, in‐stent restenosis; NSTEMI, non‐ST‐segment elevation myocardial infarction; STEMI, ST‐segment elevation myocardial infarction
**Figure S2** Distribution of QFR values (A) Distribution of individual QFRs values before and after DCB angioplasty. (B) Rate of vessels with VOCE according to different QFR strata after DCB angioplasty. DCB, drug‐coated balloon; QFR, quantitative flow ratio; VOCE, vessel‐oriented composite endpoint

## Data Availability

The data that support the findings of this study are available from the corresponding author upon reasonable request.
