# Supplementary material for: Clinical implication of quantitative flow ratio to predict clinical events after drug‐coated balloon angioplasty in patients with in‐stent restenosis
Source: Clin Cardiol. 2021 May 19;44(7):978–86. doi: 10.1002/clc.23630 (PMC8259159; doi:10.1002/clc.23630)
Supplement: Supplementary file 1 — Table S1 Baseline patient characteristics (n = 177) Figure S1 Flow chart of patient selection. DCB, drug‐coated balloon; ISR, in‐stent restenosis; NSTEMI, non‐ST‐segment elevation myocardial infarction; STEMI, ST‐segment elevation myocardial infarction Figure S2 Distribution of QFR values (A) Distribution of individual QFRs values before and after DCB angioplasty. (B) Rate of vessels with VOCE according to different QFR strata after DCB angioplasty. DCB, drug‐coated balloon; QFR, quantitative flow ratio; VOCE, vessel‐oriented composite endpoint [file CLC-44-978-s001.docx]

| **Table S1** Baseline patient characteristics (n = 177) | | | |
| --- | --- | --- | --- |
|  | **VOCE** | **Non-VOCE** | ***p* value** |
|  | **(n = 26)** | **(n = 151)** |  |
| Age, years | 66 (63–73) | 69 (62–75) | .883 |
| Male, n (%) | 18 (69.2) | 125 (82.8) | .177 |
| Hypertension, n (%) | 20 (76.9) | 111 (73.5) | .714 |
| Diabetes mellitus, n (%) | 18 (69.2) | 66 (43.7) | **.016** |
| Insulin-treated, n (%) | 7 (26.9) | 31 (20.5) | .463 |
| Hyperlipidemia, n (%) | 7 (26.9) | 28 (18.5) | .322 |
| Previous or current smoker, n (%) | 11 (42.3) | 86 (56.9) | .166 |
| Previous myocardial infarction, n (%) | 8 (30.8) | 47 (31.1) | .971 |
| Previous PCI of non-target lesion, n (%) | 6 (23.1) | 35 (23.2) | .991 |
| Time to restenosis, days | 490 (290–1966) | 321 (210–1723) | .120 |
| Left ventricular ejection fraction, % | 63 (60–69) | 63 (59–68) | .661 |
| Clinical features |  |  |  |
| Stable angina pectoris, n (%) | 15 (57.7) | 92 (60.9) | .755 |
| Unstable angina pectoris, n (%) | 6 (23.1) | 45 (29.8) | .484 |
| NSTEMI, n (%) | 5 (19.2) | 14 (9.3) | .130 |
| Multivessel disease, n (%) | 17 (65.4) | 103 (68.2) | .776 |
| eGFR, mL/min/1.73 m^2^ | 62.1 (38.7–73.9) | 67 (57.3–80.4) | .060 |
| LDL-c, mmol/L | 2.17 (1.90–2.72) | 2.04 (1.67–2.73) | .596 |
| HbA1c, % | 6.7 (6.2–7.9) | 6.4 (5.9–7.6) | .149 |
| Medication at baseline |  |  |  |
| Aspirin, n (%) | 23 (88.5) | 142 (94.0) | .533 |
| Clopidogrel, n (%) | 15 (57.7) | 85 (56.3) | .894 |
| Ticagrelor, n (%) | 11 (42.3) | 66 (43.7) | .894 |
| *P* values < .05 are in bold.  *Note:* Values are shown as median (25th–75th percentile) or number (%).  Abbreviations: eGFR, estimated glomerular filtration rate; HbA1c, glycosylated hemoglobin; LDL-c, low-density lipoprotein cholesterol; NSTEMI: non-ST-segment elevation myocardial infarction; PCI, percutaneous coronary intervention. | | | |

| **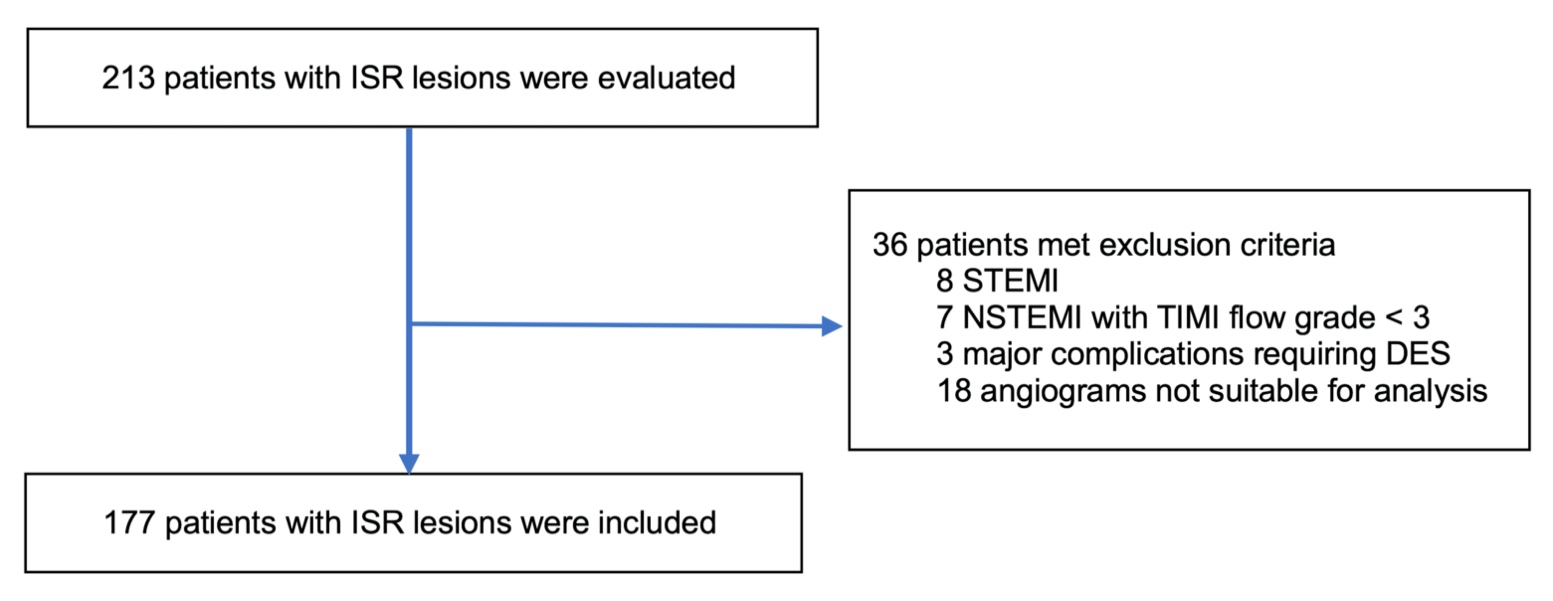** |
| --- |
| **Figure S1** Flow chart of patient selection. DCB, drug-coated balloon; ISR, in-stent restenosis; NSTEMI, non-ST-segment elevation myocardial infarction; STEMI, ST-segment elevation myocardial infarction. |

| **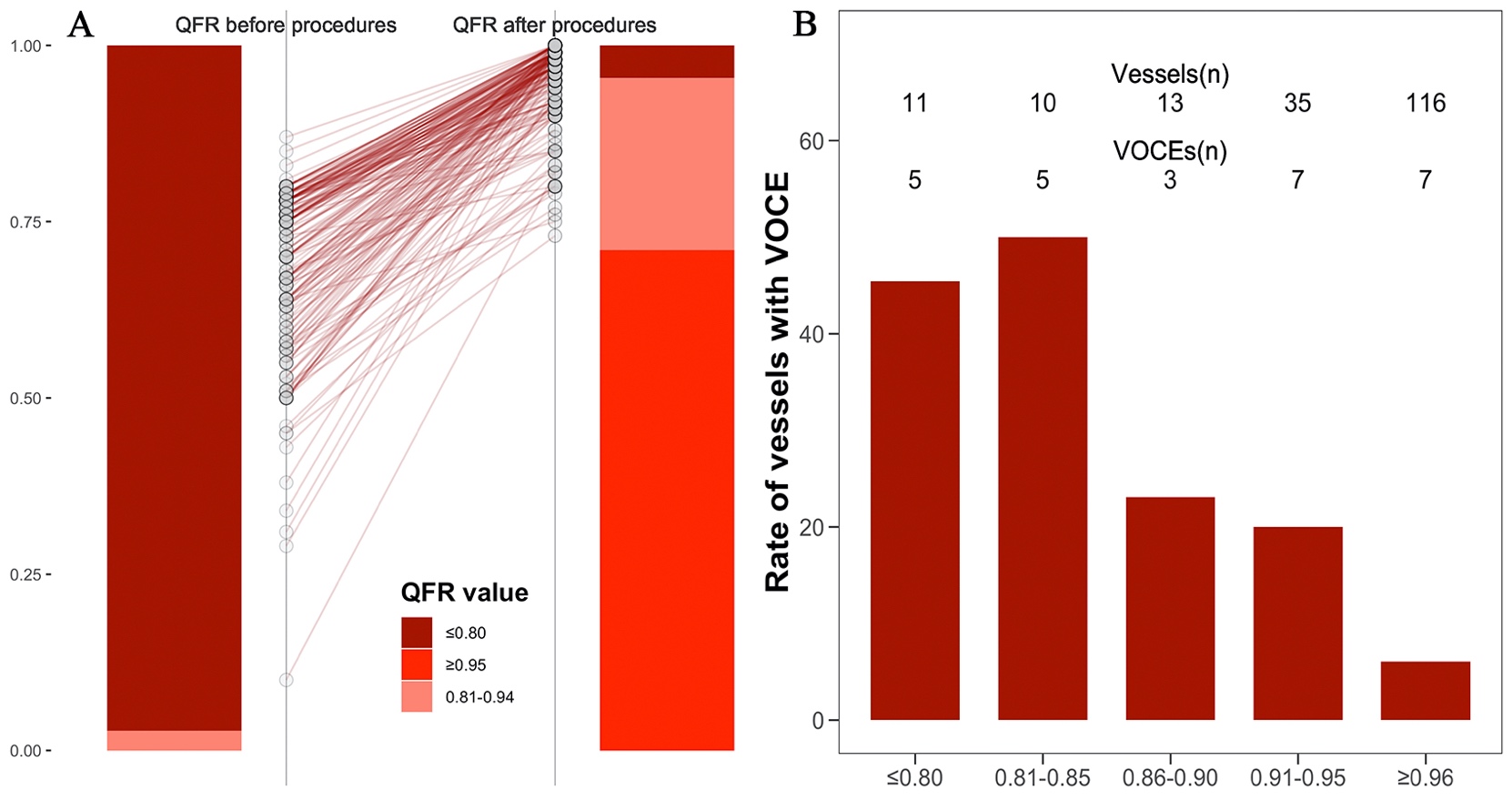** |
| --- |
| **Figure S2** Distribution of QFR values A) Distribution of individual QFRs values before and after DCB angioplasty. B) Rate of vessels with VOCE according to different QFR strata after DCB angioplasty. DCB, drug-coated balloon; QFR, quantitative flow ratio; VOCE, vessel-oriented composite endpoint. |
